# Recent Advances of Point-of-Care Devices Integrated with Molecularly Imprinted Polymers-Based Biosensors: From Biomolecule Sensing Design to Intraoral Fluid Testing

**DOI:** 10.3390/bios12030136

**Published:** 2022-02-22

**Authors:** Rowoon Park, Sangheon Jeon, Jeonghwa Jeong, Shin-Young Park, Dong-Wook Han, Suck Won Hong

**Affiliations:** 1 Department of Cogno-Mechatronics Engineering, Pusan National University, Busan 46241, Korea; rowoon.p153@gmail.com (R.P.); jsfhse@pusan.ac.kr (S.J.); j_purification@pusan.ac.kr (J.J.); nanohan@pusan.ac.kr (D.-W.H.); 2 Department of Dental Education and Dental Research Institute, School of Dentistry, Seoul National University, Seoul 03080, Korea; nalby99@snu.ac.kr; 3 Department of Optics and Mechatronics Engineering, Pusan National University, Busan 46241, Korea

**Keywords:** molecularly imprinted polymer, point-of-care test, biomolecule, oral disease, wearable device

## Abstract

Recent developments of point-of-care testing (POCT) and in vitro diagnostic medical devices have provided analytical capabilities and reliable diagnostic results for rapid access at or near the patient’s location. Nevertheless, the challenges of reliable diagnosis still remain an important factor in actual clinical trials before on-site medical treatment and making clinical decisions. New classes of POCT devices depict precise diagnostic technologies that can detect biomarkers in biofluids such as sweat, tears, saliva or urine. The introduction of a novel molecularly imprinted polymer (MIP) system as an artificial bioreceptor for the POCT devices could be one of the emerging candidates to improve the analytical performance along with physicochemical stability when used in harsh environments. Here, we review the potential availability of MIP-based biorecognition systems as custom artificial receptors with high selectivity and chemical affinity for specific molecules. Further developments to the progress of advanced MIP technology for biomolecule recognition are introduced. Finally, to improve the POCT-based diagnostic system, we summarized the perspectives for high expandability to MIP-based periodontal diagnosis and the future directions of MIP-based biosensors as a wearable format.

## 1. Introduction

Molecular diagnostics point-of-care (POC) is a technology belonging to the field of personalized healthcare and refers to clinical pathology tests for the diagnosis of disease [1]. Generally, it has been used to enhance the therapeutic effect by enabling an immediate test next to the patient, which was tested in the field of immunology and clinical chemistry [2]. POC devices are a type of in vitro diagnostic (IVD) medical device, designed for the purpose of diagnosing various diseases to determine prognosis, evaluating health status by medical treatment effect and even preventing disease [3]. The market growth for IVD devices can be attributed to the increasing proportion of the geriatric population and technological advancement in diagnostics [4]. Recently, during the COVID-19 pandemic, growing interest in public healthcare has rapidly boosted up rapid testing kits, anticipating the development of various types of devices with market expansion. Although the expensive cost of product development may defer research demand for POC testing (POCT), some progressive technologies are continuously introduced by merging existing portable sensing platforms in line with recently developed bioelectronic devices with suitable configurations in medical diagnosis applications [5,6,7]. One aspect of bioelectronics is the application of physicochemical signal transducers to detect substances at the molecular level and recognize interactions through signal processing. Biosensors used in POCT encompass a wide range of topics for the detection of analytes, various types of receptors, such as enzymes, antibodies, antigens, proteins at the interface of biological molecules and sensors. Therefore, since the POCT can be performed in close proximity to the location where the patient is being treated, emerging technologies as a potential alternative may replace the conventionally used laboratory-based diagnostic testing, including different combinations of components for sample handling and recognition elements. Thus far, the recent trend in the integration of diagnostic devices has moved to cost-effective programable tools for rapid and sensitive detection of biomarkers in biofluids, such as sweat, tear, saliva and urine [8,9,10]. However, many biomarkers in biological samples (i.e., biological fluids) are often present at very limited concentrations, coexisting with unwanted interfering species. Therefore, the detection of biomarkers usually requires highly qualified antibodies for sensitivity detection techniques together with sampling purification. To analyze one type of biomarker, enzyme-linked immunosorbent assay (ELISA) is a widely used immunological assay in diagnostic research [11], which provides quantitative data on specific proteins in serum samples. Despite its high specificity and low limit of detection (LOD), some drawbacks still arise from relatively long procedures with moderate reliability or expensive bioassay kits’ specified protocols, depending on the primarily designed binding affinity for different targets [12].

In this context, as a rational synthetic strategy and biomimetic design in the field of biotechnology, molecularly imprinted polymers (MIPs) have been revisited in response to the continuous demand for rapid, accurate and cost-effective analytical platforms. MIPs, crosslinked polymer matrices with molecular recognition sites formed by synthesizing in the presence of a target template, have received immense attention to guarantee affordable detection modules for target molecules for decades [13,14,15]. Historically, although viewed as an old material system, the MIP technology has progressed with a renewed field of research and expanded the area by combining emerging nanomaterials and advanced detection techniques with new applications [16,17,18]. Specifically, MIPs can be considered synthetic chemocavities or antibodies, as tailor-made artificial receptors that recognize and bind target molecules with high selectivity and chemical affinity [19,20,21]. The MIP matrices can be synthesized simply by polymerization of monomers forming a complex with target molecules, in which a relatively weak bonding was set between the template molecules and crosslinked monomers. In detail, starting with the prepolymer/template mixture, the spatial arrangement of MIPs can be determined by several well-known interactions, such as hydrogen bonds, Van der Waals forces, hydrophobic interactions and electrostatic interactions [22,23,24]. Subsequently, after the removal of template molecules from the crosslinked polymeric matrices, copious cavities with specified chemical end groups can be easily crafted, depending on complemental templates defined by size, shape and chemical functionality. Indeed, a large number of results on MIP techniques have reported newly developed molecular imprinting strategies with small molecules, such as sugars, steroids, pesticides, epitopes and amino acid derivatives [25,26,27]. These previous elaborated works have demonstrated the reliable capabilities of MIPs in highly targetable recognition systems on specific molecules, used in chemical sensors, analytic separations, solid-phase extractions, drug delivery systems, catalysts and library screening methods [28,29,30].

Having been progressively specialized in the field of biotechnology, MIPs were successfully commercialized for the solid phase of drugs and pesticides for an extraction toolbox to rebind small molecules toward improved sample refinement of chromatographic analysis [31,32]. In addition, molecular imprinting for other larger-scale substances in particular is also considered a candidate for expandible technology and remains under development with plenty of potential. Biomacromolecules, including antibodies, viruses, proteins, enzymes, nucleic acids and even living cells, can readily be imprinted in precisely designed polymer matrices with the help of other interfacial molecules or additives. However, the biomacromolecule approaches in the MIP system will confront serial problems with less reliability in the sophisticated capturing process because the classical bulk methodologies for target templates may fail to precisely recognize the protein target molecules. The lack of accessibility lies primarily in the intrinsic properties of protein molecules themselves. Complete rebinding may be difficult due to imprinted sites that differ from the original conformation by irreversible structural reconfiguration during polymerization [33]. In other words, templated proteins embedded in the polymerized matrix can be partially immobilized with strong physical bonds in the crosslinked polymeric network during the template removal step, which provides fewer rebinding sites [34]. Thus, improved techniques are needed, especially in protein imprinting processes, to prevent irreversible entrapment in 3D polymeric networks. Hence, the large number of uncontrolled interaction sites by the imprinted proteins may lead to cross-reactivity on the originally provided cavities along with non-specific adsorption. Biomacromolecule recognition systems have now become a new growing research area in MIP approaches, and the control of the biological environment associated with an appropriated function (i.e., artificial recognizable antibody) requires further development in practical use and biomedical diagnostic applications [35].

In view of the above, the MIP-based pseudo-immunoassays may be developed and may strengthen the biosensing capability and related POCT to measure the concentration of small and macromolecules with the help of specialized ‘artificial antibodies’ to antigen counterparts [36]. Thus, an enhanced accuracy of MIP-based POCT will accelerate the molecular diagnostic and has critical potential to play an important role in analytical tests in various fields with different macromolecular targets for more accurate results. For now, commonly used biosensors are mostly based on the detection of antigen–antibody interactions, which are evaluated and quantified with respect to each proposed transducing mechanism [37,38,39]. Moreover, most antibodies used are proteins, which are physically, chemically and biochemically unstable for use in a medical-grade immunoassay. Therefore, healthcare devices based on MIP-technology-based POCT may provide suitable access for specific patients by providing reliable results from artificial antibody-integrated biosensors (i.e., MIP-based testing). The MIP-based biosensing platform can be robust, sensitive and accurate to enable label-free detection of biomolecular analytes. Such beneficial embodiments will meet endless supply demands in the segmental market to develop MIP technology, leading to an integration of cost-effective portable POCT with an expansion of the overall industrial progression.

In this review, we will highlight the recent progress of MIP technology, particularly with a wide range of examples of POCT-based biomarker detection. Because the biomolecules used in developing diagnostic POCT are generally complex in nature, integrating the test kits (i.e., cartridges) with appropriate antibodies requires a highly sophisticated coupling between the two components [40]. We believe that the existing problems of POCT can be solved by utilizing novel MIP technology through the sensitive detection of biomarkers in the sampling of biological fluids extracted from sweat, tears and urine, and directly allowing the detection of biomolecules [41,42,43]. However, to be commercialized for POCT, the MIP-based biosensors must overcome several challenges related to non-specific rebinding, cross-reactivity, acceptable analytical performance and practical use models. Moreover, due to continuous exposure to the fluid of interest simultaneously with other biological components, the biosensors targeting analytes may need recalibration to correct for signal responses over time. On this issue, at the end of this review, we will also suggest wearable POCT devices and advanced MIP-based technology to determine periodontal diagnostics by identifying oral fluid-based biomarkers for precision oral medicine. Since periodontitis is a complex and multifactorial disease, prognostic progression is hardly detected, subject even to chronic conditions [44]. The discovered biomarkers that cause oral disease can be molecularly imprinted on conductive polymer surfaces. With easy accessibility from saliva sampling, the MIP-based periodontic diagnosis to improve POCT-based diagnostic systems is still under investigation for clinical application, which will be an important field of study and highly beneficial for overall public healthcare.

## 2. MIPs for Biomolecule Recognition: Concepts of POCT and Synthetic Approaches

### 2.1. Concepts of the MIP-Technology-Based Portable POCT Devices

**Figure 1:**
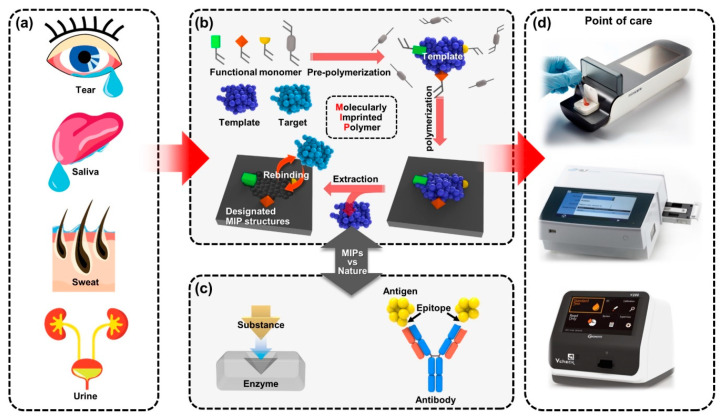
A new class of benchtop-scale POCT devices utilized by MIP-based biosensors for precision diagnostic technology to detect biomarkers in biofluids. (**a**) The four types of representative human biofluid reflecting health conditions; tears, saliva, sweat and urine. (**b**) Schematic illustration for fabricating the molecular imprinting system that contains the biorecognition sites and (**c**) the example of natural biorecognition system; enzyme–substrate complex (**left**) and antigen–antibody reaction (**right**); the biomimetic functional similarity of the MIP biosensing system is comparable to natural antibodies. (**d**) Various types of immunoassay-based benchtop-scale POCT devices.

The most important feature for MIP-enabled biosensors is the comparability that can be integrated with the existing systems, which provides high recognition ability [45]. The persistent durability allows MIPs to be used in various types of POCT applications, depending on the types of samples to be tested, as presented in Figure 1. Benchtop-scale POCT devices, incorporating MIP-based biosensors, are poised to transform the healthcare device platforms. Conventional biosensors combining biological elements have been produced in a form of chips, disposable strips, cartridges or electrodes in the application of POC devices [46,47]. However, in certain situations, the diagnostic devices have some limitations in new classes of POC devices [48]. For example, the short shelf life of biomolecular immobilized biosensors is less cost effective for manufactured products because they should be refrigerated in transport and storage [49]. Another potential issue might cause the low activity of biomolecular functions under harsh chemical conditions in some cases of biofluids or sampling, such as extreme changes in pH, saline or highly reactive solvents in certain treatment controls for the POCT devices [50], which critically affect the performance of biosensors by fundamental degradation of the disease-specific biomarkers [51]. Besides, although conventionally used bioreceptors are suitable for achieving selectivity, multiple processing with technical difficulties and delicate interface control are required in the implementation onto biosensors [52]. Since the immobilization of recognition sites is essential to transduce the signals for the operation of biosensors, advantageous materials and alternative strategies will be needed to resolve the shortcomings linked with conventional biomarker integration while achieving selectivity. By the aforementioned motivations, MIP techniques have been progressed for biosensor applications with carefully contrived design. The MIP-based biosensor can be considered an artificial antibody-integrated polymeric active layer that readily sustains stability in challenging testing chemical environments, such as high-temperature limits up to ~300 °C [53,54]. Since general proteins are usually denatured in irreversible forms higher than ~80 °C [55,56], MIP-based biosensors are more stable in storage and even suitable for applications requiring a high-temperature range. In the scene of biomolecule imprinting with low-cost materials, by taking advantage of the MIP technique to mimic biological sensing elements, such as antibodies and bioreceptors [57], a variety of single-target biosensors can be developed for diagnostic biosensors and assays for POCT devices [58]. Because the MIP-enabled biosensors extracted out the biological antibodies or other elements, the desired receptor surface can be tailored for relatively high selectivity and specificity. Compared to the biosensors integrated with natural antibodies, MIP-based biosensors have exhibited a comparable or even decreased limit of detection (LOD) with signal-to-noise enhancement and improved stability resulting in potential use for biosensing platforms [59]. The continued progress of MIP technology holds great potential with innovative key aspects of inexpensive, rapid and sensitive detection for desired POCT systems, providing other opportunities in demanding medical environments.

### 2.2. Biomolecule Imprinted Polymers Based on Bulk Imprinting Techniques

At the beginning of the development of synthetic process for MIPs, target-oriented techniques, capable of recognizing and binding biomacromolecules (e.g., proteins), have been introduced along with practical use in numerous applications, including clinical diagnostics [60], drug delivery systems [61], proteomics and environmental analysis [62]. Despite the widespread research work on MIPs, there have been some limitations in the design of the MIP material system in protein detection by the intrinsic conditions of templated proteins, such as size, complexity and structural instability. Recently, however, the synthetic strategies for protein-based MIPs have been extensively developed to improve the selective recognition capability, named by bulk, suspension, emulsion and epitope imprinting, depending on the materials mainly featured [63,64,65]. As one synthetic process for protein-based MIPs, the so-called bulk imprinting polymerization is the most commonly accepted method with apparent advantage of the simplicity of the processing scheme. In designing bulk imprinting, by using the crosslinker and the functional monomer, protein molecules can be imprinted entirely on the growing polymer matrix with randomly distributed configuration, and subsequent extraction of the templates from the MIPs complete the process with high yield performance. Finally, for the collected MIP powders, the mechanical grinding process separates the bulk-imprinted polymers into the form of micron-scale particles or beads. This sequential process suggests a viable route to produce a large number of bulk MIP particles that can be used in several commercial applications [66]. However, for a typical bulk MIP system, a random free diffusion of the templates (i.e., small molecules) was subjected to the formation of microcavities in the densely networked MIP structures [67]. Therefore, bulk imprinting is adaptable for imprinting for small molecules because the adsorption/release of templated molecules is easily expected and represents relatively fast and reversible interactions, which add value to the small-molecule imprinted matrix as multiple-time reusable assay in cost-effective benefits [68]. On the other hand, a limited synthetic condition for the bulk-imprinted biomacromolecules has been reported due to partially trapped templated molecules in the polymer chains, commonly featured with a complex distribution in the mixed state prior to the polymerization. Such drawback lies in the limited diffusion rate of biomacromolecules from the nature of bulk MIP manufacturing system. Consequently, low diffusion rates attenuate the quality of MIPs with lacking accessibility on the rebinding sites. Moreover, the mechanical grinding process as a final stage is strictly controlled to maintain the original integrity of the prepared samples, that is, damages of the recognition sites reduce the adsorption capacity of the bulk MIP system. Although crushing films into smaller dimensional microparticles notably expands binding recognition sites, the irregular shapes and sizes of the resulting bulk MIP particles lead to less reliable signal detection for accurate biosensing of biomacromolecules with high precision [69]. Thus, to avoid the instability of protein-imprinted bulk MIPs, one key parameter can be the homogeneous combination between template sizes (i.e., the large size of proteins) of the incorporated monomers by careful design to provide protein recognition sites with high reproducibility. The nanoscale protein-imprinting polymer in uniform 3D bulk scale is inevitable for an improved binding site accessibility, meeting quality requirements for biosensor application on the appropriate analytical performance [70]. The advances of protein-imprinting technique have also expanded to direct construction of micro/nanoscale surface-imprinted MIP systems on planar surfaces with the development of combinatorial materials composition, which has suggested novel sensing mechanisms in various types of signal transducing systems [71]. In competition with the bulk MIP technique, other strategic templating processes have been introduced as alternative methods to resolve the problems with diffusion limitations, uniform features and improved selectivity.

### 2.3. Biomolecule-Imprinted Polymers Based on Surface Imprinting Techniques

**Figure 2:**
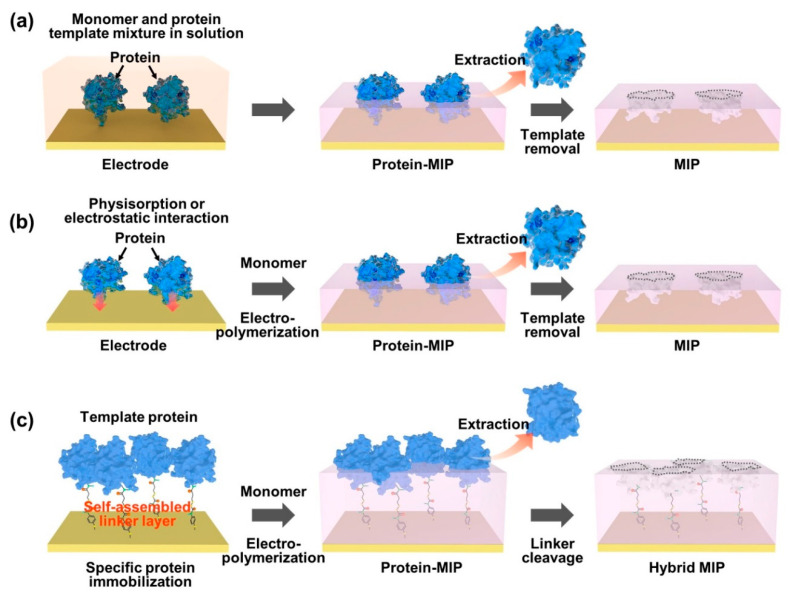
Representative strategies on the surface imprinting process to construct specific protein recognition cavities. In an appropriate design concept, the selective rebinding site can be generated by using a functional monomer for electropolymerization on a prepared electrode surface, which includes the formation of the pre-polymerization complex (**a**), the template physisorption (**b**) and the immobilization of the target protein (**c**).

As an effective way of integrating biomolecule-imprinted polymer into biosensor systems, newly developed surface imprinting techniques have been directly applied to transducing elements, such as chemically derived electric signals [72,73,74]. By the advantageous feature of the surface MIP system, the increased specific binding sites are exposed only on the surface of the polymer matrix for effective recognition, which thus accelerates mass transfer and accurate rebinding capacity (i.e., adsorption/desorption efficiency). To generate a protein-imprinted polymeric surface, a suitable monomer selection for the templates is a crucial factor in the rational design through high affinity of chemical composition for the advanced surface MIP system. Similar to the bulk MIP system, since the binding strength and stability between monomer and template depend on non-covalent weak forces, such as hydrogen/hydrophobic or electrostatic interactions, designing a template/monomer complex on the active surface area to effectively recognize the rebinding biomolecules is necessary. As shown in Figure 2, the interactions between proteins and monomers for constructing protein-imprinted polymers (i.e., artificial receptor formations) on the electrically conductive surface can be classified into several types: (i) formation of a prepolymerized complex on the electrode; (ii) sequential electropolymerization of functional monomers after template physisorption; and (iii) immobilization of the target protein using a complemental linker. As an easily accessible process, protein–monomer mixtures were introduced onto electrode surfaces using drop casting [75], spin casting [76] and spray coating [77]. After that, the prepolymerized complex was crosslinked under specific electrosynthesis conditions, and the templated protein could be extracted by physical or chemical methods to form steric cavities, constructing a surface-MIP recognition system (Figure 2a). On the other hand, Figure 2b represents another developed surface imprinting method that induces spontaneous adsorption of proteins to the electrode surface to increase templated cavities. The templates (i.e., proteins) built on the electrode surface can be imprinted by physicochemical interactions following the electrosynthetic process, in which the configured specific cavities are mostly on the surface of the MIP matrix. During this electropolymerization approach, the isoelectric point (pI) of the protein can be considered an important factor in designing sophisticated MIP–protein complexes. As an inherent property of proteins (i.e., amphiphilicity), the pI is usually defined by the pH value of a solution, at which the net charge is zero [78]. Thus, the electrostatic behavior of proteins with pH is subtle in the process because when the pH of a solution is higher than the pI value, the surface of the protein becomes predominantly negatively charged, resulting in a repulsive force on the same charged molecules. In contrast, in the case of lower pH of the solution than the pI value, the protein surface can be positively charged. However, under conditions with a pH value close to the pI, the attractive force prevails between the proteins by balancing the negative and positive charges, leading to the aggregation or precipitation of the protein [79,80]. To resolve this problem, the surface-MIP approach using a sacrificial carrier was demonstrated. By the fact that the aggregated form of proteins can lead to inadequate sensing properties in MIP-based biosensor systems, the formation of covalently immobilized proteins prior to electrodeposition of functional monomers on the electrode surface could obviously enhance the sensing performance for target proteins (Figure 2c). As one clear demonstration, the sequential molecular imprinting process of specific protein binding sites for selective recognition system in the surface-MIP structure is as follows [81]. First, the electrode surface could be chemically modified via a 4-ATP/DTSSP linker system and immobilized with a target protein (CDNF). After electrochemical polymerization with functional monomers, selective molecular cavities could be formed simply by the S–S bond cleavage process. This experimental approach implies that target protein immobilization can be facilitated by a simple combination of conventional chemistries (i.e., linkers such as self-assembled monolayers, SAMs) to create more uniform specific binding sites with finely tuned affinity [82,83,84], instead of random electropolymerization from the protein/monomer mixture. Notably, in this case, the optimized thickness of the surface-MIP film was precisely controlled during the electropolymerization process not to exceed the height of the immobilized target protein, which is the most important factor in avoiding irreversible entrapment of proteins in the imprinted polymer matrix [85].

### 2.4. Electrosynthetic Strategies for Biomolecule-Imprinted Polymers

**Figure 3:**
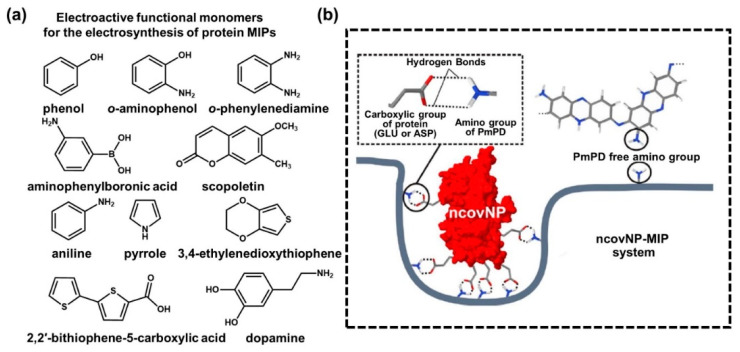
(**a**) Representative electroactive monomers used for implementation of the MIP-based biorecognition system; chemical structures of each functional monomer are listed as follows: phenol, o-aminophenol, o-phenylenediamine, aminophenylboronic acid, scopoletin, aniline, pyrrole, 3,4-ethylenedioxythiophene, 2,2′-bithiophene-5-carboxylic acid and dopamine. (**b**) Schematic illustration of the ncovNP–MIP system to form multiple non-covalent bonds between ncovNP and cavity. Reproduced with permission from [80]. Copyright Elsevier, 2020.

As described in the previous section, direct electropolymerization has proved to be an efficient technique to construct surface-imprinted MIPs by the adequate combination of monomers and templates [86,87,88]. More importantly, the selection of biomolecular templates has not been limited within an allowed experimental condition and is ready to be applicable to state-of-the-art electropolymerization strategies on the electrically conductive electrode surfaces, such as RNAs, DNAs, peptides, aptamers, antibodies, proteins, viruses, bacteria and even living cells [89,90,91,92,93,94,95,96]. A suitable choice of monomers and templates plays a key role in the molecular design of electrodeposition techniques with delicate modulation of parameters for the MIP-enabled conducting polymer matrix. Thus, synergistic influences on surface MIPs have been evaluated as a result of highly specified analytical performance in the biosensing platforms [97,98]. As reported earlier, the main parameters for electrosynthetic process can be summarized as follows [99]: (i) voltage or current of applied potential; (ii) potential scan rate and periodic potential pulses during deposition cycles; and (iii) the restriction of electrical density on the electrode. Therefore, the electrosynthesis of conductive polymers in the surface-MIP system highly depends on the series of optimization by a control of the surface morphology, density and film thickness to tune the capability of charge transfer passing through the electrode [100]. Figure 3a represents a typical process of electropolymerization for protein-imprinted polymers. In the protein-imprinted polymerization step of the electrosynthesis process, the stacking monomer layers and boundaries define the shape of the complemental recognition sites according to the size of biomolecules, such as proteins with embedded functional groups. Thus, sequentially designed processing steps with tailored compositions can be important to define the exposed areas of the cavities and controlled distributions of biomolecules prior to the electrodeposition of monomers. Such electroactive monomers on a prepared electrode surface to be grown as an electrically conductive polymeric matrix should be carefully selected according to the electrosynthesis conditions because there are many combinatorial options with other binding assistant chemicals, including phenol, o-aminophenol, o-phenylenediamine, aminophenyl boronic acid, scopoletin, aniline, pyrrole, 3,4-ethylene dioxythiophene, 2,2′-bithiophene-5-carboxylic acid and dopamine, as previously reported [101,102,103,104]. For example, as illustrated in Figure 3b, a variety of combinations has been demonstrated by using o-phenylenediamine (o-PD) on template proteins for the surface-MIP integration [105,106]. Moreover, based on a computational approach, Raziq et al. recently demonstrated that o-PD could be a reasonable choice for biologically active MIPs compared to a set of molecules, such 3,4-ethylenedioxythiophen (EDOT) and dopamine (DA), in binding to the SARS-CoV-2 viral protein [107]. Looking into the detailed performance, the Glide empirical scoring function (GScore) values for the scoring binding pose of other monomers (i.e., o-PD, dopamine and EDOT) docked to SARS-CoV-2 nucleoprotein (ncovNP) were similar, in the ranges of −25.2 and −29.5 kJ mol^−1^, confirming that they could form stable pre-polymerized complexes with ncovNP. As a result of the quantum chemical calculation, the H-bond interactions on the ncovNP molecules adjunct with NH_2_ groups were determined decisively; the o-PD monomer was found to be a superior option compared to other monomers. This computational modeling approach can be highly useful and expanded to the advanced designing of MIPs, especially for the electrosynthetic process, because the molecular reaction and energy startup guidance based on the predominant parametric assumptions might be derived without repetitive control experiments [108]. In the biomolecular imprinting field, this high-end computational approach may be advantageous in rapid development in choosing correct parameters between the template and functional monomer to realize MIP-based biosensors, yielding highly selective recognition sites by scrutinizing the critical interaction energies [109]. By doing this, the electrosynthetic strategies for viral-protein-based MIPs (i.e., SARS-CoV-2) can contribute to producing a new concept of POCT devices to fully utilize the electrically operational transducers [110], which is under development with the popularly introduced small-scale device integrated with microchips for the wearable or skin-attachable format [111]. We will discuss this in more detail in the following sections.

## 3. Transducing Systems and Practical Approaches for MIP-Based Biosensors

### 3.1. Mass Sensing Approaches

**Figure 4:**
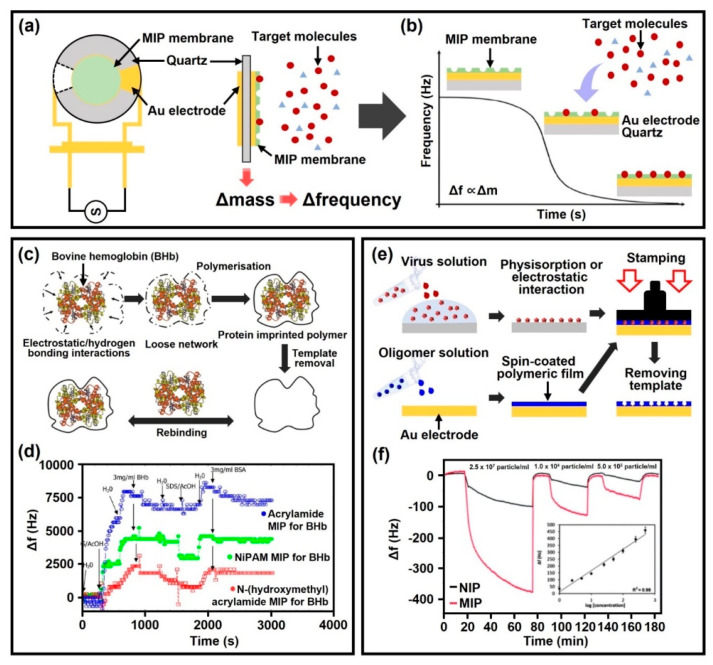
(**a**) A conceptual design for an MIP-based QCM sensor capable of selective adsorption of target molecules. (**b**) Frequency change in QCM transducer according to mass change due to binding between the target molecules and artificial receptors formed on the MIP membrane. (**c**) A synthetic strategy to prepare hydrogel-based MIPs for BHb protein adsorption. Reproduced with permission from [119]. Copyright The royal society of chemistry, 2014. (**d**) Frequency response of polyacrylamide-based MIPs to BHb and BSA protein. Reproduced with permission from [119]. Copyright The royal society of chemistry, 2014. (**e**) Schematic illustration of a molecular imprinting process using template stamp coated with influenza virus. (**f**) Frequency changes of MIP- and NIP-based QCM sensor at different concentrations of H1N3 influenza A virus; the inset shows a change in the frequency according to virus concentration (r^2^ = 0.98). Reproduced with permission from [114]. Copyright The royal society of chemistry, 2013.

To date, MIP-based quartz crystal microbalance (QCM) sensors have been regarded as one of the most promising sensing techniques and have been developed as an analytical method to detect various biomolecules, such as DNA, peptide and viral protein [112,113,114]. In a typical configuration of a QCM-based sensor, an AT-cut quartz wafer with metal electrodes on either side is coupled with an oscillator circuit that drives the QCM to resonate at an its intrinsic frequency. According to the basic principle of QCM operation, an analyte adsorbed on the electrode surface causes a change in the fundamental resonant frequency of the crystal, which precisely determines the mass of the adsorbed analyte, that is, the resonant frequency is decreased depending on loaded mass [115]. Because MIP membranes contain specific molecular recognition sites with size, shape and chemical features complementary to the desired target molecule, the combination of a QCM sensor and MIP membrane with artificial receptors can selectively recognize target molecules in complex biological samples and minimize cross-sensitivity with sufficient susceptibility (Figure 4a). Therefore, as biomolecules are adsorbed into the cavity of the MIP membrane, the fundamental resonant frequency gradually decreases in the MIP-based QCM sensor system (Figure 4b). The technical parameter for imprinting small molecules or metastable biologicals lies in the selection of functional monomers and appropriate solvents for MIP synthesis. For example, small molecules are stable in organic solvents, whereas biomolecules are unstable and can be denatured under similar conditions [116]. Therefore, many efforts have been made to construct water-soluble polymer systems, such as hydrogels for imprinting biomolecules [117,118].

As illustrated in Figure 4c, a conceptual demonstration was performed as a QCM-based diagnostic system using molecularly imprinted hydrogels to detect bovine hemoglobin (BHb) [119]. By the optimized synthesis condition for the hydrogel-based MIP membrane with specific binding capacity, three distinct types of the acrylamide functional monomer were utilized, such as acrylamide (AA), N-hydroxymethylacrylamide (NHMA) and N-isopropylacrylamide (NiPAm). The graph in Figure 4d describes the gradual QCM-based frequency shifts through the sequential immersion process in BHb, SDS/AcOH and bovine serum albumin (BSA) solutions; the molecular weight of BSA and BHb corresponds to 66.5 kDa and 64.5 kDa, respectively. The degree of cross-selectivity for non-target proteins (i.e., BSA) revealed the recognition site formation (i.e., cavities) with protein complemental to the target (i.e., BHB) in a hydrogel-based MIP system. In the BHb-imprinted MIPs formed by AA, NHMA and NIPAM-based matrices, an apparent decrease in resonant frequency by the BHb adsorption process was confirmed without changes in additional BSA proteins. In particular, as a hydrogel-based MIP system, the detection performance for BHb was correlated with the degree of hydrophilicity and increased in the order of polyNHMA, polyacrylamide and polyNIPAM. Consequently, the high selectivity of NHMA–MIP for the BHb protein was observed by the presence of a hydroxyl group in the cavity architecture. Although AA-based MIP was equally selective for both BHb and BSA proteins, the absence of hydroxyl groups in the cavities derived a relatively weak ability to distinguish between cognate and non-cognate proteins.

As another outstanding example of QCM-based biomolecules detection system, Figure 4e shows the selective capture of infectious influenza A (i.e., H1N3, H1N1, H5N3, H5N1 and H6N1 viruses) by combining MIPs and QCM-based gravimetric transducer [120]. To prepare the MIP-based QCM sensor, a delicate sequential process was used utilizing an MIP precursor solution containing functional monomer, cross-linking agent and a photoinitiator. The MIP solution was spin-casted on an Au electrode, and then a stamp coated with the template (i.e., virus stamp) was pressed onto the spin-coated MIP prepolymer film. In this experimental scheme, acrylamide (AMM), methacrylic acid (MAA), methylmethacrylate (MMA) and N-vinylpyrrolidone (VP) were used as functional monomers. In the following, MIP prepolymer film incorporating virus particles was polymerized under a UV light source with 254 nm wavelength overnight, and the template (i.e., virus) was extracted by denaturing the virus from the polymeric network by using 10% hydrochloric acid. As a result, the limitation of selective recognition of virus subtypes was dramatically enhanced due to the addition of the VP monomer. The specificity and sensitivity for the template were further improved by controlling the ratio between the different monomers and the cross-linking agent.

Figure 4f shows a response curve for the QCM-based MIP sensor capable of detecting the H1N3 influenza A virus over time. The calibration curve showed that the logarithmic relationship between frequency and virus concentrations responds to mass changes in a linear form (r^2^ = 0.98). When equilibrium is reached between the MIP membrane and the surrounding solution, the MIP signals increase at least 5-fold compared to non-imprinted polymer (NIPs) signals. In the case of NIPs, it is desirable that specific binding to the target molecule be restricted, but this is not achievable in practice due to unpredictable and non-specific adsorption or physical influences between the NIP membrane and the target molecule. Accordingly, the QCM sensing system integrated with an MIP membrane that contains a memory effect from the template molecules (i.e., recognition sites) provides high affinity to the target virus in a reproducible manner for more reasonable molecular detecting tools. In this way, the MIP-coated QCM sensor can be expected in the next generation of molecular sensor platform to support diagnostic POCT to evaluate small proteins, artificial enzymes or viruses.

### 3.2. Electrochemical Sensing Approaches

For the MIP-based biosensors, to evaluate the complemental interactions between the analytes and cavities (i.e., receptors) formed on the electrode surface, the analytical signals should be transduced and converted into a quantitative range of values [120]. On this, we first focus on the electrochemical sensing approach, which is quite suitable for MIP-based biosensors with various types of electrode configurations to validate a rebinding of the analytes [121]. Due to ease of access, biomolecule analytes can be measured quantitatively, combined with external redox materials in the solution-type tests. The measured value changes are originated from the Faraday current, corresponding to the redox reactions on the MIP-based electrode in cyclic voltammetry (CV) or differential pulse voltammetry (DPV) [122]. By well-known basic fundamental ideas, the mechanism, caused by the diffusion of the redox probe, is usually understood in specific operating conditions for the MIP-based electrochemical sensors [123,124]. For example, the physical docking of analytes (e.g., viruses, small molecules and proteins) into the surface cavities can generally block the diffusion of the redox probe on the MIP electrode surface in amenable chemical reactions [125,126].

**Figure 5:**
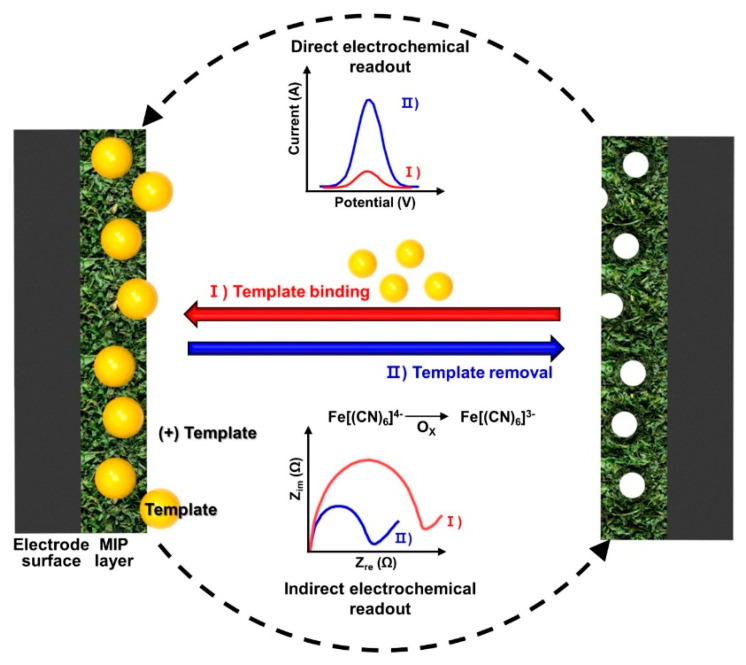
The basic concept of the electrochemical transduction on the MIP-based sensors using voltammetry test; the sensing procedure requires typical sequential steps, including the removal of the templates and rebinding of the target analytes on the MIP electrode. An electrochemical analytical method can be selected depending on the electroactive property of the analytes, which can be categorized either through direct detection (i.e., direct electrochemical readout) of the analyte or indirect detection (i.e., indirect electrochemical readout) using a redox probe, such as [Fe(CN)6]^3/4−^.

Figure 5 illustrates the basic concept of electrochemical transduction on MIP-based sensors. The sensing procedure requires sequential steps, including rebinding of the target analytes on the MIP electrode and washing to remove the non-specific binding elements. Using voltammetry test, the detection of analytes on MIP-based electrochemical methods can be performed to evaluate the current density according to the potential range for waveform techniques (e.g., differential pulse, square wave, linear sweep or staircase) [127]. On the basis of this technical support, the advantages of the voltammetry test for MIP-based biosensors are prompt analyses to determine the selectivity and sensitivity of the engaged analytes by electrochemical reacting on the electrode within a given concentration range. Among the voltammetry test, the DPV technique is the most acceptable technique for MIP-based biosensors, with relatively simple implementation and a low level of noise by the capacitive current. Amperometric sensing approach by measuring the generated current at the sensing electrode surface with respect to a fixed time interval can be an effective method at a constant single-potential step, known as the chronoamperometric technique. This amperometric method directly reflected the measured current with the concentration of the analytes at a constant applied potential; therefore, the analytes are detected by the facilitation of the built-in MIP electrode. The signal-changing value collected from the amperometric device is of an apparent reading gauge, interfaced with the MIP-based electrode, since the mass transfer rate was delivered from the signal-changing value by electrochemically active analytes. Electrochemical impedance spectroscopy (EIS) is a sensitive characterization method of collecting the electrical signals from chemical responses, in which the time response with the chemical systems was susceptible based on low-amplitude alternating current and voltages over a range of frequencies. Therefore, the quantitative values can be obtained by this technique, but the chemical mechanisms for the localized conditions at the electrode surface and electrolyte solution would be more complicated depending on the analytes [123,127]. However, the EIS system covers a wide range of MIP-based sensing matters as an effective analytical tool to find analytic characterization for biosensor transductions. The following Table 1 summarizes recently presented works on electrochemical sensors that detect various molecules.

### 3.3. Practical Uses of MIP-Based Biosensors: Urgent Demand and Immediate Contribution

Disposable POC diagnostic devices have supported the patient-centered healthcare system. With rapid signal acquisition, some device platforms are convenient and cost effective in assisting clinicians and patients with reasonable diagnostic coverage. However, in some cases, these diagnostic devices have inevitable limitations, such as short shelf life to keep the sensors refrigerated in transport and storage because of the antibodies and receptors embedded in the disposable kits. Another potential issue can concern the chemical conditions when performing analyte detection by changing the conditions of pH, saltwater or highly reactive organic solvents, as previously described. Notably, as an innovative artificial ‘plastic’ receptor, the MIP-based POC diagnostic platform offers high stability and can be stored at room temperature, operating in challenging physical and chemical environments to expand its applications.

**Figure 6:**
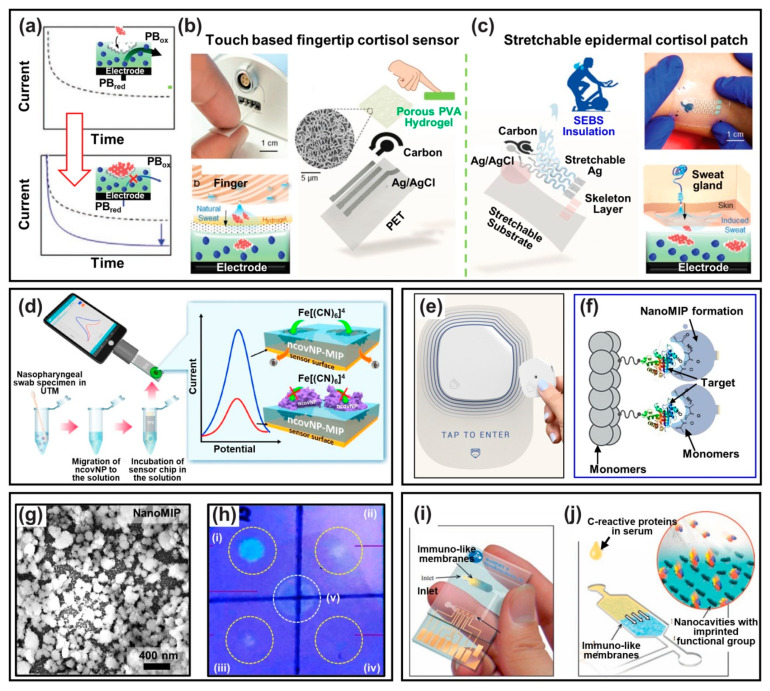
Disposable POC diagnostic biosensors. (**a**) The stress-free sensing platform to detect endogenous cortisol levels from the sweat sampling in an electrode structure with a porous MIP membrane; the reduced current can be measured by oxidation of the imprinted PB by cortisol rebinding process. Reproduced with permission from [133]. Copyright Wiley–VCH, 2021. (**b**) Schematic diagram of MIP-based cortisol sensor based on ‘touch/incubate/detect’ protocol. Reproduced with permission from [133]. Copyright Wiley–VCH, 2021. (**c**) A demonstration of the MIP-based biosensor for a conformal epidermal-integrated patch that tracks changes in cortisol levels during on-body exercise. Reproduced with permission from [133]. Copyright Wiley–VCH, 2021. (**d**) A mobile COVID-19 diagnostic system equipped with a disposable MIP-based biosensor. Reproduced with permission from [106]. Copyright Elsevier, 2021. (**e**,**f**) An image and concept of commercialized COVID-19 biosensor produced by solid-phase polymerization for nanoMIP to capture SARS-CoV-2. (**g**) SEM image of the nanoMIP film surface. (**h**) Optical micrographs captured from the dot blot assay for SARS-CoV-2 detection; (i) SARS-CoV-2 full-length spike protein trimer (positive control), (ii,iii) test position (2 × 10^4^ PFU), (vi) viral culture media only (negative control), (v) CPN 510B (reference signal). (**i**,**j**) Scheme of the MIP-based POCT device based on immuno-polymeric membranes with a confined fluidic flow and defined electrode array to isolate CRPs in human serum samples. Reproduced with permission from [128]. Copyright Elsevier, 2013.

As one example of the disposable POC diagnostic platform, Figure 6a illustrates recently reported MIP-based biosensor by utilizing a typical three-terminal electrode, in which the MIP working electrode was prepared by an electrochemical synthesis and plated with a porous membrane (PVA hydrogel) to impregnate PB (Prussian blue) redox probe. In this configuration, endogenous cortisol levels were detected from the sweat sampling. In this approach, a cheap screen-printed electrode (SPE) was covered with a porous MIP membrane containing a PB redox probe. This biosensor can readily detect the exposed cortisol analytes in sweat upon fingertip palpation through a specific rebinding on a cortisol-imprinted polymeric membrane (i.e., PVA hydrogel). The MIP membrane was found to be sensitive to the amperometric method by providing engraved cavities from the PB probe. Compared to the previous methods, such ‘touch/incubate/detect’ protocol is innovative in the development of the POC device, highlighted by rapid detection time (~3.5 min) in quantifying cortisol levels simply from a fingertip based on the current changes (Figure 6b). Since cortisol is linked to mental health, monitoring cortisol levels will be an important indicator of early detection of psychological conditions. The highly permeable liquid-absorbing porous membrane, according to a capillary reaction of sweat, can be extended to other examples of MIP-based biosensors to instantaneously collect other secreted biofluids from the human body [134,137,138]. Besides, the authors also demonstrated the expandability of MIP-based biosensors to a conformal epidermal-integrated patch by tracking changes in cortisol levels during on-body exercise, showing excellent performance after repeated use (60 cycles) for real-time cortisol monitoring (Figure 6c). The recent development of healthcare systems already moves to skin-attachable devices or even implantable electronic circuits [139,140,141], and thus, MIP-based biosensors will be able to be securely integrated with other platforms for potential application to meet the required high selectivity.

The worldwide pandemic situation by an infectious coronavirus disease (COVID-19), caused by the SARS-CoV-2 virus, accelerated the realistic MIP-based biosensing system [107,136,142]. Since rapid detection by using an MIP-based electronic device provides an easily accessible and stress-free approach, a portable electrochemical biosensor can obviously be useful for POC tests when molecularly imprinted with a SARS-CoV-2 nucleoprotein (i.e., ncovNP-MIP). As an example, a synthetic recognition element integrated biosensor for selective detection of ncovNP was recently reported, as presented in Figure 6d [143]. In their demonstration, the MIP-based COVID-19 sensor showed linear responses to the cavities from ncovNP in the apparent concentration range of 2.22–111 fM in the lysis buffer; within the measured values, LOD and LOQ were valued as 15 and 50 fM, respectively. In this straightforward approach, the ncovNP-imprinted biosensor could transduce the signals from the specific rebinding of the ncovNP in nasopharyngeal swab samples collected from COVID-19-positive patients. This demonstration implies that the MIP-based virus sensors have a great potential to extend their application to other infectious mutant viruses for rapid diagnostic tools as POCT kits [144]. Referring to the recently reported COVID-19 POCT with a detection performance within ~2 min (LOD: ~5 fg mL^−1^), it may be an efficient assessment to simply measure current changes through optimized settings in the MIP-integrated electrode [136].

As a similar MIP approach in this categorized application, MIP-based POCT outperforms commercially used antibodies for a biosensor platform by revealing an improved detection capability with lower viral loads during an extended infection period (Figure 6e) [138]. By a designed polymerization process with SARS-CoV-2, precise molecular imprinting could be successfully performed for the receptor-binding domain (RBD) region by mimicking the spike glycoprotein (i.e., target), as described in Figure 6f. A SEM image in Figure 6g shows the surface morphology of the crafted *nanoMIPs* (i.e., nanoparticle-featured surface imprinting). In this experiment, a solid-phase imprinting process was used to promote intimate chemical interactions between the template and functional monomers with stoichiometric chemical moieties during the immobilization process (Figure 6h). For detecting SARS-CoV-2, the nanoMIP was combined with fluorescent polymeric nanoparticles (FPNs) to visualize the virus recognition capability simply by using a dot blot assay, as shown in Figure 6i; these FPN-integrated MIPs yielded significantly brighter signals (i.e., 10,000 times higher level) than other samples [145]. The measured areas coated with nanoMIP film are shown as follows: (i) positive controls for SARS-CoV-2 spike protein; (ii) and (iii) a SARS-CoV-2 capture region; (iv) negative control with virus culture medium only; v) reference control. The imaged dot blot arrays were able to selectively detect SARS-CoV-2 and reported a low LOD value of 5 fg mL^−2^. By further selectivity evaluation, the SARS-CoV-2-imprinted biosensing platform only recognized SARS-CoV-2 spike glycoprotein in the dot blot assay, whereas no responses with the human coronavirus spike glycoprotein (299E, HKU1, OC43) were detected. Supported by a reliable scale-up manufacturing process, this manipulated nanoMIP platform may give rise to an impact on the regular diagnosis for quick check-up of COVID-19 in hospitals, drive-through sites or at home, as an effective POCT kit. Indeed, the progressive type of nanoparticle-based MIPs (i.e., nanoMIP for a single species) could extend their POCT applications to other target molecules, such as enzymes or proteins, because the system provides more selective and specific rebinding sites for high accuracy in diagnostic testing.

Figure 6i displays a novel MIP-based POCT device for protein recognition based on an immune-polymeric membrane used to isolate C-reactive proteins (CRPs) from serum samples. In their approach, the cavities structured in the MIP-integrated membrane were combined with a confined fluidic flow, interlocked on a defined electrode array. In particular, the biosensing performance was evaluated by the separation principle in a critically aligned configuration of CRPs on the working electrode, as drawn in Figure 6j. By this setting, the impedance changes were detected directly on the applied current, responding to the CRP rebinding reaction in the MIP-integrated membrane. Rapid detection of CRPs was evaluated within 2 min, starting with incubation of serum samples. Their biomimetic immuno-membrane manifests several advantages in the MIP-based biosensor technology by rendering receptors as biological sensing elements. Therefore, the electrochemical detection method is compatible with the structured MIP membrane that is addressed in the defined sensing area. With regard to its high compatibility with microfabrication processes, it is possible that other advanced techniques can be applied to 3D nanoporous vertical channels to engineer high specificity.

## 4. Concept of Oral POCT to Detect Diseases: Novel Detection in Salivary Biomarkers

The advantage of the user-friendly POCT as a wearable form is perfectly fit for new diagnostic concepts by detecting small molecules from the collected biofluid sampling, since that process does not require specialists or complicated treatment with medical equipment [134]. As is well known, saliva includes tremendous biomarkers, including substances secreted from salivary glands, external substances, microorganisms and blood-derived compounds, reflecting oral diseases or systemic diseases [146,147,148]. However, given the low concentration of biomarkers in saliva, effective detection can easily lead to false signals by contamination of external factors [149]. However, the continuous interest in molecule sensing from saliva has extended the research area in wearable device applications, from which in situ saliva analysis has been rapidly developed. Thus, several intuitive ideas have been suggested to minimize the contamination of saliva sample, divided mainly into a mouthguard platform for direct measurement of biomarkers from saliva in the oral cavity or external sensing with a microfluidic system as IVD devices. In this final section, we summarize the recent development of wearable oral biosensing devices for detecting a set of biomarkers in saliva and conclude with the proposal of MIP-integrated biosensing platform as a promising approach in the same categorized study.

**Figure 7:**
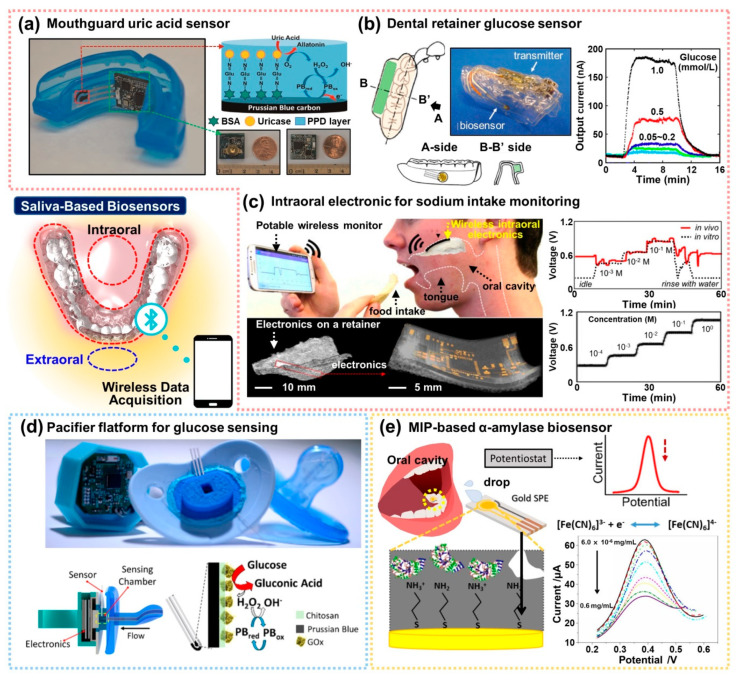
Concept of non-invasive biosensing platforms mounted in the oral cavity for real-time monitoring by wireless communication to detect molecules (middle left image). (**a**) A mouthguard integrated with a wireless circuit board for monitoring salivary UA and the chemically modified SPE sensor for electrochemical detection. Reproduced with permission from [150]. Copyright Elsevier, 2015. (**b**) Customized glucose biosensor mounted on a plastic retainer with a detection range of 0.05–1.0 mM L^−1^ in artificial saliva. Reproduced with permission from [153]. Copyright Elsevier, 2016. (**c**) A wireless electronic device mounted on a retainer with a permeable porous membrane that detects sodium ions. The graphs show clear changes in the electrical signals according to sodium concentration based on in vitro and in vivo experiments. Reproduced with permission from [155]. Copyright National academy of sciences, 2018. (**d**) A pacifier-type glucose biosensor for external monitoring of saliva collected without any pump through the fluidic channel. Reproduced with permission from [158]. Copyright American Chemical Society, 2019. (**e**) Scheme of α-amylase imprinted biosensing platform on Au-SPE electrode to quantify the concentration range of 6.0 × 10^−6^–0.6 mg mL^−1^ through the electrochemical method. Reproduced with permission from [83].

Biosensors mounted on mouthguards are straightforward as one good example of the POCT approach. Recently, as shown in Figure 7a, Kim et al. presented an integrated wireless mouthguard to sense salivary metabolites based on an amperometric sensing platform to detect uric acid (UA) in diluted saliva for smart healthcare monitoring [150]. The amperometric enzyme electrode is the oldest platform since the first introduction of the glucose biosensor by Clark in 1962. Briefly, the detection of ion presence on solution based on electric current or changes in electric current has been called ‘amperometry’. As addressed in Section 3.1, an amperometric biosensor induces a current proportional to the concentration of the substance to be detected. In line with this electrochemical approach for a practical wearable device, the wireless mouthguard biosensor was integrated with a Bluetooth-enabled circuit board, built on flexible PET (polyethylene terephthalate) substrate. A biosensor embedded as a wearable mouthguard was firstly demonstrated by utilizing an SPE transducer in a flexible format and mounted in mouthguard preform [151]. In detail, they used a simple chemical modification on the commonly used screen-printed working electrode by electropolymerized oPD and simultaneous crosslinking of the uricase enzyme. Therefore, a soft mouthguard was facilitated for continuous monitoring of UA in saliva. The wearable device was configured with an amperometric transducer and coupled by wireless communication systems (i.e., Bluetooth), which were readily integrated into a system on chip as a singular product. The saliva sample could be collected from the mouth for real-time sensing, and the current signals were extracted in the continuous operation for 4 h in the monitoring process with 10 min intervals with a stability of the electrochemical response of 300 mM UA. Within the optimized experimental condition, they reported a current response of every 0.5 s at a 2 Hz frequency with a sensitivity of 2.45 mA mM^−1^. Accordingly, the result from the wireless mouthguard type salivary UA sensor enabled the transfer of data measured from real-time detection. This new concept of biosensors offered an attractive electrochemical sensing platform with high sensitivity and selectivity. The ‘in mouth’ mounting in the human body still requires a critical assessment of biocompatibility with less toxic materials that are essential for the realization of wearable electronic devices [152].

As a similar approach presented in Figure 7b, a customized mouthguard-type biosensor was also introduced by Arakawa et al. [153]. This wearable oral POCT, so-called ‘cavity sensor’, was produced on a plastic substrate (i.e., polyethylene terephthalate glycol, PETG) to be a mountable oral cavity for non-invasive saliva analysis. The presented biosensor consisted of Pt, Ag/AgCl and glucose oxidase (GOD) electrodes, immobilized by poly (MPC-co-EHMA) (PMEH) for glucose monitoring. With these configured electrodes, the output current produced by glucose oxidation at GOD was measured by the amperometric method as a function of the concentration of H_2_O_2_. The characteristic sensing performance in artificial saliva was set for 1.0 wt% PMEH matrix with an electrode surface area of 16.8 mm^2^ to measure the glucose, showing high selectivity in current output only for glucose, compared to other saliva analytes, 100 mM L^−1^ of galactose, sorbitol, fructose, mannitol and xylitol solution. Moreover, this GOD-based biosensor exhibited high sensitivity in PBS and artificial saliva ranging from 0.05 to 1.0 mM L^−1^ under a wireless condition in continuous real-time data collection. Therefore, advanced oral biosensors will be helpful in non-invasive monitoring systems for diabetic patients. Although there may be differences in glucose levels between blood and saliva samples, a personalized POCT device for glucose sensing can be a promising approach for further development into a nontoxic and safe mouthguard-type platform [154].

Another alternative to in vivo oral monitoring devices has been introduced to estimate sodium intake. As illustrated in Figure 7c, Lee et al. presented a novel biosensor with a customized dental brace composed of a biocompatible elastomer that can measure sodium ion concentration in direct contact with the oral cavity [155]. The active electrode for the biosensor was fully embedded in a microporous structured elastomer (i.e., Soma Foama 15, SF15) to package an integrated circuit system equipped with stretchable interconnects. Thus, the hybrid form of bioelectronic device enabled conformal contact with intraoral tissues. Owing to the high permeability based on the breathable SF15 membrane, the sodium ion sensor is perfectly suited for detecting sodium intake in the oral cavity with high selectivity and sensitivity. By optimizing impedance matching to the circuits incorporated with the porous membrane, real-time quantification of sodium intake was realized for wireless data transfer. This sodium sensor in the oral cavity showed clear changes of electrical signals as a function of the sodium concentration ranges, such as 10^−4^, 10^−3^, 10^−2^, 10^−1^ and 1 M, under in vitro experimental condition, demonstrating high sensitivity and selectivity; the level of output voltage was also compatible with the result from in vitro experimental detection capability. In particular, soft electronics with user-friendly interface can take advantage of favorable physical properties to actively collect information about not only sodium intake but also dietary habits and health management [156].

In general, the geometry of the oral cavity varies from person to person. Thus, an externally configured sensing element may be preferred when direct contact in the mouth is limited due to the lack of teeth or the discomfort that would permit using biosensors [157]. As proof of concept, a biosensor-embedded pacifier was designed for wearable oral POCT devices to monitor the glucose concentration levels in saliva, as shown in Figure 7d [158]. In this demonstration, the pacifier made from nontoxic silicone served a useful function as a fluidic saliva collector, integrated with an SPE-based biosensor for electrochemical detection, in which an amperometric circuit was connected with a miniaturized wireless data transfer module. Since the presented POCT device contained a saliva-fluidic channel (i.e., biofluid reservoir), this unique design helped the biosensor operation by soaking the exposed working electrode in unidirectional saliva flow without any suction pressure, allowing the sensor to detect glucose based on the glucose oxidase modified PB transducer. The electrochemical method with functional electrode offered good selectivity for glucose with no responses to 200 μM of UA and 20 μM of ascorbic acid (AA) and without any interfering crosstalk from substances left in the mouth. When the sensitivity was scanned, the current changes in glucose concentration in artificial saliva exhibited a well-defined ranged concentration between 0.1 and 1.4 mM. The PB-oxidase electrode proved to be suitable for monitoring glucose concentration by monitoring the correlation between blood and saliva glucose before and after food intake. Aside from the instability of the enzyme-based electrode and incomplete sterilization with each use, the only minor limit of the pump-free pacifier-based sensing platform was the delayed duration of saliva collection time to reach the exposed working electrode with signal stabilization. However, assisted by this progressive work, additional study on the choices of effective design and more efficient instrumentation of microfluidic channels will pave the way for a viable route for the development of wearable oral POCT.

In this section, we have discussed various approaches for salivary biomarker detecting biosensors and PCOT devices in a wearable format. As a similar but slightly different approach, MIP technology as artificial receptors is ready to be combined together with the intraoral POCT devices and amperometric molecular recognition system. Although it has not yet been reported in this field of research, when the MIP system can be directly applied to a biosensing system, especially in an oral disease monitoring system, it could have far-reaching implications and clearly stand out by resolving some drawbacks with improved performance. Since some approaches on the protein-based MIP on the SPE surface have been reported previously for detecting salivary protein as a combinatorial result of the electrochemical method [159,160,161], it is easily possible to transform the strategies on the bioactive electrode preparation and the selection of suitable materials for nontoxic packaging. A recent α-amylase imprinted biosensor is a representative example of stress-related healthcare monitoring for potential POCT (Figure 7e). In this case, a typical amperometric transducer was used for the ranged quantification of the α-amylase concentration by the utilization of Au-SPE electrodes, at which the surface of the working electrode was electropolymerized with conductive pyrrole and α-amylase. With an accurately controlled Au surface using a cysteamine self-assembled monolayer, the α-amylase template was effectively immobilized. In this MIP process, the biomarker (i.e., α-amylase) can leave behind highly specific cavities after the removal of the templates in the polypyrrole matrix on the electrode (refer to Section 2.4). Next, a typical electroanalysis was conducted using one of the pulse techniques, that is, square wave voltammetry (SWV) [162]. In this discriminated sensing, the MIP biosensor to capture a specific molecule from oral biofluid represented outstanding performance in the α-amylase concentration range from 6.0 × 10^−6^ to 0.60 mg mL^−1^ in buffer solution with high sensitivity (LOC < 3.0 × 10^−4^ mg mL^−1^). Due to the nature of the MIP-based sensing system [163], it showed high sensitivity and selectivity through the rebinding process only on target biomolecules in human saliva and in a buffer solution containing other biomarkers. Conclusively, this MIP biosensor exhibited analytical capabilities as a promising candidate for diagnostic POCT devices when integrated with sophisticated electrodes and a real-time wireless system [83].

## 5. Conclusions and Outlook

Due to the outbreak of COVID-19, there has been an increasing interest in technology and product developments in the field of molecular diagnostic POCT with the highest accuracy and rapid identification. From a long-term perspective, significant technological progress for on-site medical treatment will persist, and a new format of POC devices and related techniques will be introduced to the market due to the change in work methods caused by the COVID-19 and the generalization of telecommuting. In this context, this review discussed and summarized the engineering innovations of POCT devices equipped with MIP-based biosensing systems. In particular, the integration of MIP-based electrochemical biosensors with POCT-based diagnostic systems can be expected to be adaptable in IVD devices for an accurate and selective detection of biomarkers caused by various diseases, not only for viral proteins.

Up to date, there has been a large set of advances in developing various types of MIP-based POC devices in biomedical diagnostics for emergency assessment. The detection of biomarkers at trace levels in biofluids using the MIP-based biosensing device may provide highly qualified artificial receptors for biologically specific and selective recognition together with an easy manufacturing process. Therefore, with the strong demand for non-invasive and rapid evaluation, the use of MIP-based biosensors offers new opportunities in disease identification and risk-resolving assessment by managing the fundamental preservation of disease-specific biomarker-imprinted cavities in POC devices, free from shelf life, cost effectiveness and storage issues. In addition, in the categorized research field, the detection of biomarkers in periodontology remains limited overall. Artificial antibody-based approaches highlighted in this review imply great potential in dental care and medicine. Because chronic immune-inflammatory responses to microbial biofilms formed in the oral tissue or dental implanted surface are in systemic conditions, such as cardiovascular disease, diabetes or gastrointestinal diseases, the detection of the secreted biomarkers will be important guidelines for oral and other systemic diseases, including identifying SARS-CoV-2 in saliva [164]. Advances in electrochemical biosensing platforms with appropriate transducing systems have recently accomplished remarkable innovations in interfaced wearable electronics with biomarker assessment [110]. As a future direction, our suggested examples highlight MIP-integrated devices in oral biomarker detection and their validation for clinical POCT to advance precision medicine. This suggests that the potential of collecting clinical outcomes from wearable biometric interfaces relevant to chronic prognosis will benefit public health.

## Figures and Tables

**Table 1:** Summary of versatile approaches for detecting biomolecules using MIP-based biosensors.

	Template	Monomer	Form of MIPs	Electrochemical Techniques	Template Removal	Selectivity	LOD	Ref.
Protein	Human interleukin- -1*β* (IL-1*β*)	EriochromeBlack T (EBT)	Carbon	EIS	0.1 M PBS/CV	IgG, Myo	1.5 pM	[86]
	Human interleukin-2 (IL-2)	MAA/MBA	CdTe QDs	Fluorescence measurements	.	.	5.91 fg mL^−1^	[128]
	C-reactive proteins	O-4-nitrophenylphosphorylcholine (O-4NPPC)	Gold	Circular dichroism (CD) measurements	10% sodium dodecyl sulfate	.	.	[129]
	BSA	Raethyleneglycol diacrylate (TEGMPA), diacryloyl urea (DAU), ammonium persulphate (APS)	MWCNT	DPV	Methanol	.	.	[130]
	Thrombin	Acrylamide (AM), Methylenebisacrylamide (MBAA)	Hydrogels	Shrinking measurements	4.3 M GuCl/1.4 M NaCl.	BSA	1000 fM	[131]
Small molecule	2.4-dichlorophenoxyacetic acid (2.4-D)	PMMA (Poly(methyl methacrylate)	Gold	QCM	Acetic acid	Atrazine, Ametryn, BA	7.73 μg mL^−1^	[62]
	Formaldehyde (HCHO)	TFMAA	Gold	QCM	.	HCl, HF	24.2 and 8.0 ppm	[132]
	Chloramphenicol	EriochromeBlack T (EBT)	Laser-induced graphene (LIG)	EIS	ACN solution	Amoxicillin/clavulanic acid (AMC), oxytetracycline (OTC), sodium sulfadiazine	0.62 nM	[133]
	Cortisol	Polypyrrole (PPy)-Prussian blue (PB)	Carbon	CA	0.1 M PBS/CV	Glucose, lactate, urea, ascorbic acid, acetaminophen, uric acid	0.9 and 0.2 nM	[134]
	Caffeine	Pyrrole (PPy)	Gold	EIS	PBS/resonance frequency	Theophylline	.	[135]
Virus	SARS-CoV-2 nucleoprotein	Poly-m-phenylenediamine (PmPD)	Gold-TFE	DPV	10% acetic acid solution	S1, E2 HCV CD48 and BSA	15 fM, −50 fM	[107]
	SARS-CoV-2 nucleoprotein	Dithiothreitol (DTT)	Gold	SWV, CV	10% acetic acid	IgG, E2, HSA	15 fM −64 fM	[136]

## Data Availability

Not applicable.
